# Osteosarcoma as a communication-driven disease: redefining tumor–bone signaling networks as therapeutic targets

**DOI:** 10.3389/fonc.2026.1823738

**Published:** 2026-07-22

**Authors:** Gabriele Ricciardi, Vincenzo Fiorentino, Pietro Tralongo, Maria Lentini, Ludovica Pepe, Valeria Zuccalà, Mariagiovanna Ballato, Giuseppe Giuffrè, Antonio Ieni, Biagio Zampogna, Marco Ferlazzo, Guido Fadda, Maurizio Martini

**Affiliations:** 1 BIOMORF Department of Biomedical, Dental, Morphological and Functional Imaging Sciences, A.O.U. Policlinico “G.Martino”- Via Consolare Valeria 1, Messina, Italy; 2 Istituto Clinico Polispecialistico C.O.T. Cure Ortopediche Traumatologiche s.p.a., Messina, Italy; 3 Department of Human Pathology of Adults and Developmental Age “Gaetano Barresi,” Division of Pathology, University of Messina, Messina, Italy; 4 BIOMORF Department of Biomedical, Dental, Morphological and Functional Imaging Sciences, Section of Orthopaedic and Trauma Surgery, A.O.U. Policlinico “G.Martino”, University of Messina, Messina, Italy; 5 Operative Research Unit of Orthopaedic and Trauma Surgery, Fondazione Policlinico Universitario Campus Bio-Medico, Rome, Italy; 6 Research Unit of Orthopaedic and Trauma Surgery, Department of Medicine and Surgery, Rome, Italy

**Keywords:** extracellular vesicles, immunotherapy, intercellular communication, osteosarcoma, tumor microenvironment

## Abstract

Osteosarcoma (OS) is an aggressive primary bone malignancy characterized by high metastatic potential and limited therapeutic progress. Beyond genomic instability, accumulating evidence indicates that OS operates as a communication-driven disease in which dynamic intercellular signaling networks within the tumor microenvironment regulate tumor growth, immune evasion, metastatic dissemination, and therapy resistance. This review integrates current knowledge on immune cell crosstalk, extracellular vesicle–mediated signaling, soluble factors, and direct cell–cell interactions that coordinate local and systemic tumor progression. We discuss how these interconnected communication circuits shape the tumor immune microenvironment and sustain adaptive plasticity. Finally, we highlight emerging strategies aimed at disrupting pathological signaling networks, while acknowledging that many communication-targeted approaches remain at the preclinical stage or require further clinical validation. Framing OS as a communication-driven ecosystem provides a unifying conceptual model to identify novel therapeutic vulnerabilities.

## 1 Introduction

Osteosarcoma (OS) is the most common primary malignant bone tumor, predominantly affecting children and young adults. Despite advances in surgery and multi-agent chemotherapy, patient outcomes have remained largely unchanged over the past decades, particularly for metastatic or recurrent disease (1, 2). Lung metastasis remains the principal determinant of poor prognosis, with 5-year survival rates below 20% compared with 60–70% in localized cases (3).

Although OS is characterized by profound genomic instability, genetic alterations alone fail to fully account for its biological heterogeneity and clinical behavior (4, 5). Increasing evidence indicates that tumor progression is critically shaped by dynamic interactions between malignant cells and the tumor microenvironment. These multicellular signaling networks regulate immune escape, metastatic competence, and therapy resistance (6).

In this review, we define a communication-driven disease as a pathological condition in which disease initiation, progression, adaptation, and therapeutic response are governed not only by intrinsic alterations within malignant cells but also by dynamic and reciprocal intercellular communication networks (7). Unlike the conventional tumour–microenvironment (TME) paradigm, which primarily emphasizes the influence of surrounding stromal components on tumour behaviour, the communication-driven framework considers tumour progression as an emergent property of a highly interconnected signalling system involving malignant cells, immune populations, stromal cells, bone-resident cells, endothelial cells, and distant metastatic niches (8). Within this model, tumour cells are not simply recipients of microenvironmental cues but active participants in continuously reshaping the signalling landscape through reciprocal and adaptive communication. Accordingly, this review proposes that OS can be conceptualized within this framework and provides an integrated overview of the molecular and cellular communication mechanisms that coordinate disease progression and influence therapeutic response.

## 2 Osteosarcoma as a communication-driven disease

The concept of OS as a communication-driven disease builds upon, rather than replaces, the established paradigm of TME interactions. While the conventional TME model primarily describes the reciprocal interactions between tumour cells and their surrounding stromal components, the communication-driven framework emphasizes the organization, integration, and dynamic adaptation of signalling networks operating across multiple cellular compartments (8). Within this perspective, communication itself becomes the functional unit that ultimately drive tumour progression, immune evasion, metastasis, and therapeutic resistance emerge.

Several biological and clinical observations support this conceptual framework. First, unlike many malignancies driven by recurrent actionable oncogenic alterations, OS lacks a recurrent actionable oncogenic driver capable of consistently explaining disease behaviour across patients. Instead, it is characterised by profound genomic instability, extensive chromosomal rearrangements, and marked interpatient heterogeneity, suggesting that tumour progression cannot be adequately explained by tumour-intrinsic programmes alone (9–12). Second, accumulating evidence indicates that adaptive bidirectional communication between tumour cells and the surrounding bone, stromal, immune, and vascular compartments represents a major mechanism underlying disease progression, metastatic dissemination, and therapeutic resistance by continuously reshaping the tumour ecosystem in response to environmental and therapeutic pressures (13–18). Third, numerous preclinical studies demonstrate that disrupting key intercellular communication pathways, including cytokine and chemokine signalling, extracellular vesicle (EV)-mediated communication, and other microenvironmental signalling axes, can simultaneously impair tumour growth, modulate immune responses, reduce metastatic potential, and enhance the efficacy of conventional therapies (17, 19–21). Although direct comparative studies remain limited and broader clinical validation is still required, these findings collectively suggest that targeting communication networks may influence multiple hallmarks of OS biology more broadly than strategies directed exclusively against tumour-intrinsic pathways.

Several biological characteristics further distinguish OS from many other solid tumours and make it particularly suitable to be interpreted within this framework. OS develops within the uniquely dynamic bone microenvironment, where physiological bone remodelling depends on continuous communication among osteoblasts, osteoclasts, osteocytes, mesenchymal stromal cells, endothelial cells, and immune populations. Rather than generating entirely novel signalling pathways, malignant cells hijack and progressively reprogramme these physiological communication networks to sustain tumour growth, immune evasion, metastatic dissemination, and adaptation to therapeutic pressure (7). Consequently, the biological behaviour of OS is more appropriately understood as the emergent property of a dynamically interacting multicellular ecosystem than as the consequence of tumour cell-autonomous alterations alone.

Within this ecosystem, OS cells actively exchange signals with bone-resident cells, stromal populations, immune infiltrates, endothelial compartments, and distant metastatic niches, particularly in the lung (6, 22). Multiple and partially overlapping communication modalities operate in this network. Soluble mediators regulate paracrine and systemic signaling (23), EVs enable protected long-distance transfer of regulatory molecules (24), and direct cell–cell contacts, including adhesion complexes and tunneling structures, allow rapid signal exchange and coordinated cellular responses (25). The coexistence of these complementary communication modalities generates redundancy and adaptive flexibility, enabling OS cells to remodel the bone niche, suppress antitumour immunity, promote angiogenesis, establish metastatic sites, and dynamically adapt to therapeutic pressure (26). Collectively, these observations support the view that communication is not simply one component of OS biology, but a fundamental organising principle that integrates the diverse cellular elements of the tumour ecosystem and drives disease progression.

## 3 Molecular landscape of osteosarcoma as a driver of communication

### 3.1 Genomic instability

The molecular landscape of OS is characterized by profound structural and chromosomal instability, which extends beyond a hallmark of malignancy and actively shapes tumor–microenvironment communication (27). Unlike tumors driven predominantly by recurrent point mutations, OS exhibits extensive copy number alterations, structural rearrangements, and chromothripsis, reflecting catastrophic genomic remodeling events frequently associated with TP53 loss and RB1 pathway disruption (4, 27).

Chromothripsis generates neoantigens and promotes cytosolic DNA accumulation, leading to activation of the cGAS–STING pathway and induction of inflammatory signaling (28, 29). This establishes a direct mechanistic link between intrinsic genomic instability and modulation of innate immune communication (29).

In parallel, TP53 deficiency alters EV biogenesis and cargo composition, enriching tumor-derived vesicles with oncogenic proteins and regulatory non-coding RNAs (ncRNAs) capable of reprogramming stromal and immune cells both locally and at distant sites (30, 31).

Thus, genomic instability in OS not only drives intratumoral heterogeneity but also provides the molecular substrate for adaptive intercellular signaling, facilitating immune modulation, metastatic conditioning, and microenvironmental reprogramming.

### 3.2 Tumor-intrinsic oncogenic pathways influencing communication

Beyond structural instability, OS cells exploit oncogenic signaling networks that, although traditionally considered tumor-intrinsic, actively regulate intercellular communication by controlling cytokine secretion, EV cargo, and stromal interactions. These pathways regulate the production of soluble mediators, EV cargo, and adhesion-related signals, thereby amplifying tumor–microenvironment crosstalk.

The PI3K/AKT pathway is frequently activated in OS through PTEN loss, receptor tyrosine kinase signaling, and ncRNA-mediated regulation (32, 33). Although traditionally viewed as a tumor-intrinsic survival pathway, PI3K/AKT also shapes intercellular communication by regulating cytokine secretion, EV biogenesis, metabolic adaptation, and EMT-associated programs (34). Through these mechanisms, it enhances tumor-stromal crosstalk and promotes a microenvironment that supports invasion, metastasis, and therapy resistance (35–37). Although traditionally viewed as a tumor-intrinsic survival pathway, PI3K/AKT also shapes intercellular communication by regulating cytokine secretion, extracellular vesicle biogenesis, metabolic adaptation, and EMT-associated programs. Through these mechanisms, it enhances tumor–stromal crosstalk and promotes a microenvironment that supports invasion, metastasis, and therapy resistance. Experimental inhibition of PI3K/AKT suppresses tumor growth and restores chemosensitivity in preclinical models, highlighting this pathway as both an oncogenic driver and a regulator of communication within the tumor microenvironment (38–40).

STAT3 is constitutively activated in a substantial subset of OS, largely through IL-6/JAK signaling originating from tumor and stromal cells within the bone microenvironment (41, 42). Beyond promoting proliferation and survival, STAT3 functions as a central communication hub by integrating inflammatory signals with transcriptional programs that regulate cytokine production, EMT, angiogenesis, and immune modulation (42, 43). Persistent STAT3 activation reinforces paracrine feedback loops between OS cells and mesenchymal stromal cells, thereby sustaining metastatic progression, metabolic adaptation, and therapy resistance (42, 44, 45). Accordingly, pharmacological or genetic inhibition of STAT3 disrupts these communication circuits and reduces tumor growth and invasiveness in experimental models (46, 47).

The Wnt/β-catenin pathway is frequently dysregulated in OS and contributes to tumor progression beyond its canonical role in regulating proliferation and survival (48, 49). Aberrant Wnt signaling influences intercellular communication by promoting EMT-like plasticity, stimulating VEGF production, and enhancing extracellular matrix remodeling through matrix metalloproteinases (50, 51). These effects strengthen tumor–stromal interactions, facilitate angiogenesis, and support metastatic dissemination (52, 53). Wnt/β-catenin signaling also cooperates with inflammatory and mechanotransduction pathways to sustain adaptive communication within the tumor microenvironment (54, 55). Experimental evidence further suggests that its activation contributes to therapy resistance and stem-like phenotypes, reinforcing its role as a communication-associated regulator of tumor plasticity (56).

MDM2 amplification represents an additional tumor-intrinsic alteration that indirectly influences intercellular communication by sustaining the survival of genomically unstable OS cells (57, 58). Through functional suppression of p53, MDM2 favors persistence of stress-adapted cellular states capable of maintaining aberrant cytokine production, extracellular vesicle release, and microenvironmental signaling (59). Although its primary role remains cell-autonomous, MDM2 contributes to the establishment of communication networks that support tumor progression and adaptation to environmental stress (60, 61).

Collectively, these oncogenic pathways should not be viewed solely as cell-autonomous drivers of OS progression but as regulators of intercellular communication that coordinate signaling between tumor cells and the surrounding microenvironment. By controlling cytokine secretion, EV cargo, adhesion programs, and stromal interactions, they integrate intrinsic oncogenic alterations with multicellular communication networks that sustain immune evasion, metastatic dissemination, and therapy resistance. Subsequent sections focus on how these communication mechanisms operate across the different cellular compartments of the osteosarcoma ecosystem.

### 3.3 Tumor-intrinsic programs influencing metastatic communication

Metastatic dissemination in OS is driven by coordinated molecular programs that, while partly tumor-intrinsic, are tightly coupled to dynamic communication with distant microenvironments, particularly the lung (62, 63). Comparative genomic and transcriptomic analyses reveal substantial heterogeneity between primary tumors and matched metastases, supporting distinct evolutionary trajectories in which metastatic clones may arise from late-stage tumors or evolve in parallel during tumor progression (62, 63). However, metastatic competence in OS extends beyond genetic divergence and involves multiple adaptive biological programs, including metabolic remodeling, autophagy, hypoxia responses, cytokine signaling, and immune modulation, as described in different experimental studies (64–66). Indeed, autophagy serves as a critical survival mechanism that facilitates metabolic adaptation and resistance to microenvironmental stress in OS, acting as a context-dependent driver of tumor progression and a potential therapeutic target (67, 68). These findings highlight that metastatic competence in OS is not solely a cell-autonomous property but emerges from continuous bidirectional communication with the microenvironment.

Although OS originates from mesenchymal cells, an EMT-like plasticity program plays a pivotal role in metastatic communication (69). Rather than a classical epithelial–mesenchymal switch, OS exhibits dynamic modulation of adhesion and cytoskeletal regulators, including E-cadherin, N-cadherin, and vimentin, with EMT-associated signatures correlating with metastatic risk (70). The presence of mesenchymal markers such as N-cadherin within OS-derived EVs further links EMT programs to systemic signaling and metastatic dissemination (71). These plasticity programs are tightly regulated by ncRNAs that modulate transcription factors such as ZEB1, Twist1, and STAT3, as well as signaling pathways including Wnt/β-catenin and NOTCH, thereby reinforcing invasive phenotypes (72–74).

Mechanotransduction further integrates microenvironmental cues into metastatic signaling. The Hippo pathway effectors YAP and TAZ function as central regulators of OS plasticity by responding to extracellular matrix stiffness, cell junction remodeling, oxygen availability, and mechanical stress (75). The Hippo cascade interfaces with receptor tyrosine kinases, TGF-β, NOTCH, Wnt, GPCR, and integrin signaling, coordinating YAP/TAZ nuclear translocation and transcriptional activation through TEAD and SMAD complexes (76–78). By interacting with EMT-associated transcription factors such as SNAIL, SLUG, and ZEB1, YAP/TAZ promote acquisition of migratory and pro-metastatic phenotypes (79, 80).

Tumor–extracellular matrix (ECM) interactions constitute another critical determinant of metastatic communication. Matrix components, including collagens, fibronectin, and laminins, modulate adhesion, migration, and matrix remodeling via integrin-dependent signaling (81). Cytoskeleton-associated signaling proteins further contribute to metastatic dissemination in OS. Ezrin, a membrane–cytoskeleton linker involved in adhesion and motility, has been associated with pulmonary metastatic progression, while its pharmacological inhibition reduced invasion and lung metastasis in experimental OS models (82). Transcriptomic studies have identified matrix-associated regulators such as matrix-Gla protein (MGP), which enhances endothelial adhesion, trans-endothelial migration, and tumor cell extravasation, with elevated expression correlating with metastatic progression (83).

The CXCL12/SDF-1–CXCR4 axis represents a key chemokine-driven pathway governing lung metastasis in OS. Elevated CXCR4 expression correlates with advanced stage, pulmonary dissemination, and reduced overall survival (84, 85). CXCR4 signaling promotes proliferation, migration, and survival while inhibiting apoptosis, primarily through activation of downstream JNK and AKT pathways. Pharmacological inhibition or miR-613–mediated suppression of CXCR4 reduces tumor growth, migration, and lung colonization, underscoring its therapeutic potential (84, 85). Adding a new layer of complexity, recent spatial transcriptomic profiling by Eigenbrood et al. has revealed that the CXCR4 signaling landscape within pulmonary metastases is characterized by significant regional heterogeneity. Their study identified that while CXCL12 is predominantly localized at the tumor periphery, another CXCR4 ligand, Macrophage Migration Inhibitory Factor (MIF), is highly expressed within the intratumoral core. This distinct spatial distribution suggests that OS cells utilize different CXCR4-dependent signaling circuits depending on their precise location within the metastatic niche, potentially coordinating both immune exclusion at the border and survival signaling in the core (86). This spatial organization is closely linked to patterns of immune evasion; indeed, Ligon et al. demonstrated that metastatic OS often exhibits an “immune-excluded” phenotype, where tumor-infiltrating lymphocytes (TILs) and immunosuppressive myeloid cells are restricted to the tumor-lung interface. The concentration of checkpoint molecules like PD-L1 and LAG-3 at this invasive front correlates with poorer progression-free survival, highlighting how the spatial architecture of the metastatic niche dictates clinical outcomes (87).

Together, EMT-like plasticity, YAP/TAZ-mediated mechanotransduction, integrin-dependent ECM signaling, and chemokine-driven axes such as CXCR4 converge to form a multilayered metastatic communication network. Through integration of biochemical, mechanical, and inflammatory cues, OS cells dynamically adapt to distant microenvironments, promoting dissemination, survival, and colonization of pulmonary niches.

## 4 Major modes of intercellular communication in osteosarcoma

Intercellular communication in OS involves multiple signaling modalities that contribute to disease progression and interactions with the tumor microenvironment (88). These mechanisms operate in a coordinated manner, integrating soluble mediators, EVs, direct cell–cell contacts, and mechanical cues within the tumor niche (**Table 1**).

**Table 1:** Intercellular communication modalities in osteosarcoma.

Communication modality	Main mediators	Source cells	Target	Functional effects
Soluble factors	IL-6, TGF-β, VEGF, CXCL12, CCL2, IL-8	OS cells, TAMs, MSCs, CAFs	Tumor, immune, and endothelial cells	Proliferation, EMT-like plasticity, angiogenesis, immune suppression, metastasis
Extracellular vesicles	miRNAs, lncRNAs, proteins, metabolites	OS cells, MSCs	Stromal, immune, lung niche	Pre-metastatic niche formation, immune evasion, metabolic adaptation
Immune modulation	PD-L1, IL-10, TGF-β, chemokines	TAMs, MDSCs, DCs	T-cells, NK cells	T-cell exhaustion, macrophage polarization, immune tolerance
Tumor–bone signaling	RANKL, M-CSF, matrix growth factors	OS cells, osteoblasts	Osteoclasts, MSCs	Osteolysis, matrix remodeling, growth factor release
Direct cell–cell contact	Connexin43 channels, nanotube cargo	OS cells, stromal cells	Neighbor tumor/stroma	Signal synchronization, miRNA/organelle transfer

### 4.1 Soluble factors and cytokine signaling

**Figure 1:**
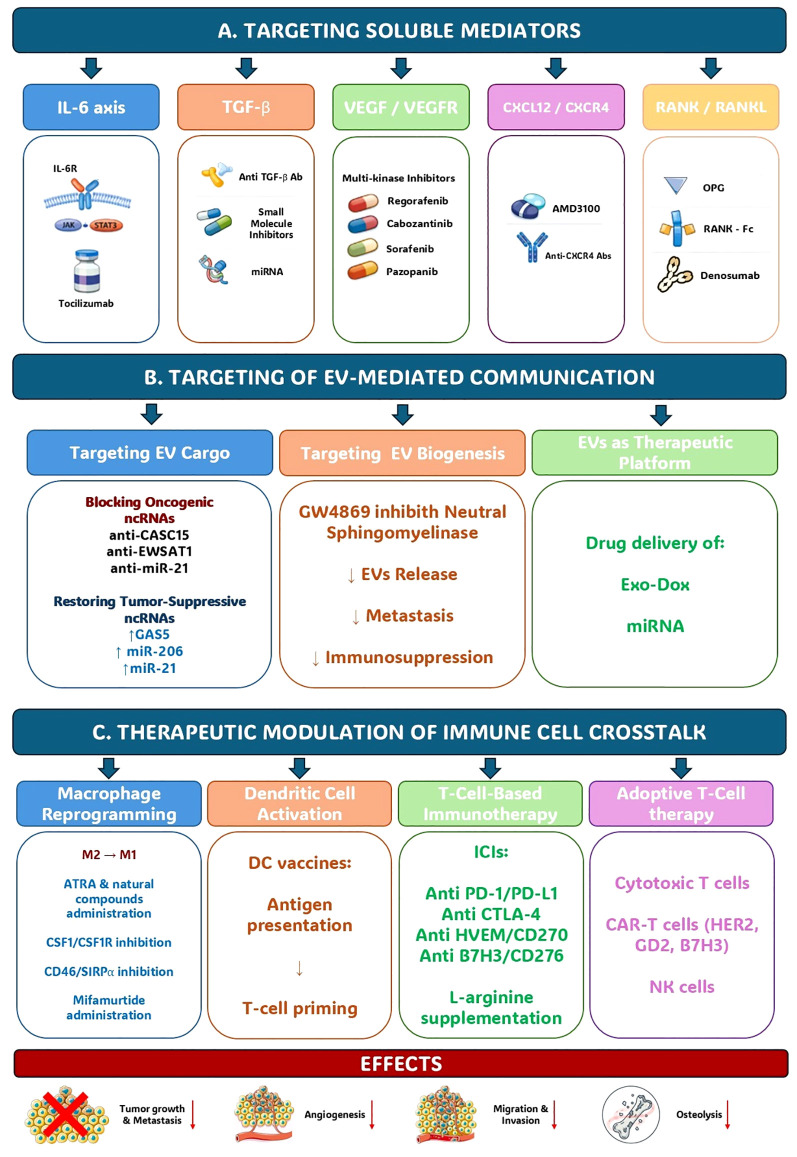
The tumor-bone vicious cycle in osteosarcoma. Schematic representation of the self-reinforcing interaction between osteosarcoma cells and the bone microenvironment. Osteosarcoma cells secrete pro-osteoclastogenic factors, including RANKL and IL-6, which stimulate osteoclast activation and bone resorption. The degradation of the bone matrix leads to the release of stored growth factors such as TGF-β and IGF-1. These factors, in turn, promote tumor cell proliferation, thereby sustaining a self-amplifying tumor–bone loop that drives disease progression.

Soluble mediators constitute a major regulatory layer of intercellular communication in OS, coordinating tumor cell plasticity, metastatic dissemination, immune modulation, and pre-metastatic niche conditioning. Tumor cells, stromal fibroblasts, osteoclasts, endothelial cells, and immune infiltrates actively exchange soluble mediators that coordinate proliferation, invasion, immune modulation, and metastatic dissemination (**Figure 1**). Among these, pro-inflammatory cytokines, growth factors, and chemokines form interconnected signaling circuits that extend from the primary bone tumor to distant metastatic sites, particularly the lung. Recent single-cell transcriptomic analyses further demonstrated that OS exhibits complex immune communication networks characterized by macrophage-driven chemokine signaling and extensive ligand–receptor interactions among immune and stromal populations within the tumor microenvironment (89).

#### 4.1.1 IL-6 axis

Interleukin-6 (IL-6) has emerged as a central integrator of inflammatory signaling and metastatic progression in OS. Aberrant ΔNp63 expression primes OS cells for increased IL-6 production; upon arrest within the pulmonary vasculature, tumor-derived IL-6 promotes micrometastatic niche formation through paracrine amplification loops and recruitment of stromal and tumor-supportive cells (90). Clinically, elevated IL-6 levels in human OS tissues correlate with advanced stage, increased proliferation, and metastatic potential (91).

Mechanistically, IL-6 enhances directional migration and invasion via upregulation of intercellular adhesion molecule-1 (ICAM-1) and activation of integrin-linked kinase (ILK), Akt, and AP-1 signaling pathways, facilitating cytoskeletal remodeling and motility (91). In parallel, IL-6 promotes proliferation and survival through STAT3 phosphorylation and SOX18 induction while suppressing apoptosis (92), thereby converging on signaling axes that overlap with the STAT3-centered inflammatory circuitry described in Section 3.2. Epigenetic regulation further amplifies this axis: TET2-dependent promoter demethylation increases IL-6 transcription, enhancing lung colonization, glycolytic reprogramming via MEK/ERK1/2–HIF-1α signaling, and ICAM-1–mediated adhesion (93). Collectively, these findings position IL-6 at the intersection of inflammatory signaling, metabolic adaptation, adhesion regulation, and metastatic competence.

Nevertheless, although mechanistic data are robust *in vitro* and in xenograft models, the relative contribution of tumor-derived versus stromal IL-6 in patient tumors remains insufficiently defined. Moreover, given redundancy within inflammatory cytokine networks, the degree to which IL-6 represents a dominant driver versus a component of a broader inflammatory program warrants further clarification.

#### 4.1.2 TGF-β signaling

Transforming growth factor-β (TGF-β) exhibits context-dependent roles in cancer and exerts multifaceted functions in OS progression. While tumor-suppressive in early oncogenesis, in advanced disease TGF-β predominantly promotes metastasis by enhancing EMT-like plasticity, invasion, angiogenesis, fibrosis, and immune evasion (94–97).

In OS, TGF-β activates canonical SMAD2/3 signaling and regulates EMT-associated transcription factors including Snail, zinc-finger E-box-binding proteins, and basic helix-loop-helix regulators (98, 99). A critical feature in OS is the bone–tumor feed-forward loop: TGF-β released during osteoclastic bone resorption stimulates tumor production of osteolytic and pro-metastatic mediators such as IL-11, connective tissue growth factor, MMP-1, CXCR4, and PTHrP, thereby amplifying bone destruction and metastatic dissemination (100, 101). TGF-β levels are elevated in OS patients and are further increased in metastatic cases, where they correlate with disease progression, poor prognosis, and chemotherapy resistance (102–104). However, the pleiotropic and stage-dependent nature of TGF-β signaling complicates therapeutic targeting, as systemic inhibition may yield context-dependent and potentially paradoxical effects.

#### 4.1.3 VEGF-mediated angiogenic and immune modulation

Vascular endothelial growth factor (VEGF) plays a pivotal role in OS development, progression, and metastatic spread. VEGF overexpression correlates with poor overall and disease-free survival, higher tumor grade, advanced stage, and increased metastatic potential (105, 106). Through activation of VEGFR2 on endothelial cells, VEGF stimulates downstream PI3K/AKT and MAPK signaling, promoting endothelial proliferation, vascularization, and increased vascular permeability, thereby facilitating tumor cell intravasation and systemic dissemination (107).

Beyond angiogenesis, VEGF modulates immune responses by inhibiting T-cell proliferation, promoting T-cell exhaustion, and recruiting immunosuppressive cell populations, contributing to immune escape (105). Clinically, VEGF overexpression is associated with poor chemotherapy response, potentially reflecting hypoxia-driven aggressiveness (105, 106). Targeting VEGF signaling with tyrosine kinase inhibitors such as sorafenib or apatinib reduces tumor growth, angiogenesis, and metastasis in preclinical and clinical contexts (108–110). Nevertheless, therapeutic responses are often partial and transient, suggesting compensatory angiogenic and pro-survival pathways.

#### 4.1.4 CXCL12/CXCR4 chemokine axis

The CXCL12/CXCR4 signaling axis is critically involved in OS progression, lung metastasis, and chemoresistance. CXCR4 expression increases with tumor grade and correlates with higher metastatic potential and poor clinical outcome (101, 111). In intratibial xenograft models, CXCR4-expressing OS cells are retained within primary bone tumors through interaction with bone marrow-derived CXCL12, limiting early dissemination (111). Conversely, in lung metastases, CXCR4 expression is markedly elevated, supporting its role in metastatic colonization and outgrowth (111).

CXCR4 activation promotes proliferation, survival, and chemotactic migration via downstream PI3K/AKT and JNK signaling pathways. Clinically, CXCR4 overexpression predicts increased local and systemic relapse (101). Pharmacological inhibition using agents such as AMD3100 and MDX1338 reduces OS cell migration, proliferation, angiogenesis, and induces apoptosis, particularly in p53-positive contexts (101). However, redundancy within chemokine signaling networks and context-dependent retention versus dissemination roles highlight the complexity of therapeutically targeting this axis.

#### 4.1.5 RANK/RANKL signaling

The RANK/RANKL axis contributes to EMT regulation and metastatic progression in OS. RANKL–RANK interaction modulates EMT-associated markers, altering E-cadherin, N-cadherin, vimentin, Snail, and Slug expression, thereby enhancing migratory and invasive capacities (112, 113). EMT transcription factors such as Snail and Slug also regulate matrix metalloproteinase expression, further promoting metastatic dissemination (113, 114).

High RANK and RANKL expression in human OS specimens correlates with poor prognosis, reduced disease-free survival, and increased lung metastasis (115, 116). Mechanistically, RANK/RANKL signaling activates NF-κB and may interact with Wnt/β-catenin pathways, reinforcing EMT and invasive phenotypes (115). Inhibition of NF-κB signaling using agents such as dimethyl fumarate (DMF) suppresses EMT and migration, suggesting therapeutic potential (115). Nonetheless, given the central role of RANK/RANKL in bone remodeling, systemic targeting requires careful consideration to avoid detrimental skeletal effects.

#### 4.1.6 CCL2 and pre-metastatic niche formation

CCL2 plays a critical role in lung metastatic niche conditioning in OS. Metastatic OS cells secrete high levels of CCL2, enhancing migration and invasion through MMP-3 upregulation mediated by suppression of miR-3659 (117). Beyond tumor-intrinsic effects, CCL2 recruits and polarizes CCR2-positive M2-like macrophages in the lung, partly via EV–associated delivery, thereby promoting pre-metastatic niche formation (118).

Neutralization of CCL2 reduces M2 macrophage accumulation and lung metastasis without significantly affecting primary tumor growth, underscoring its role in metastatic dissemination rather than tumor initiation (118). Additional mediators, including M-CSF and CXCR2 ligands, may cooperate within this axis (118). These findings identify the CCL2–CCR2 axis as a key regulator of lung metastatic niche formation and provide the biological rationale for therapeutic targeting (see Section 6.2.6).

#### 4.1.7 IL-8 and TAM-driven aggressiveness

IL-8, predominantly produced by tumor-associated macrophages (TAMs), contributes to OS progression and metastatic dissemination. Elevated IL-8 levels correlate with pulmonary metastatic sites and promote OS cell proliferation, migration, and invasion through activation of the IL-8–FAK signaling axis. IL-8 signals via CXCR1/2 receptors and can activate PI3K and PKC pathways, supporting tumor growth and metastatic competence (119).

Preclinical inhibition of IL-8 or its receptors reduces OS aggressiveness, highlighting its potential as a therapeutic target within the inflammatory tumor microenvironment (119). However, as IL-8 operates within broader inflammatory networks that overlap with IL-6 and NF-κB signaling, selective blockade may yield partial effects unless integrated into multi-target strategies.

Collectively, these soluble mediators converge on overlapping downstream pathways, including STAT3, NF-κB, PI3K/AKT, MAPK, EMT-associated transcription factors, and metabolic regulators, forming a highly interconnected and partially redundant signaling network. While individual cytokine and chemokine axes demonstrate clear mechanistic roles in metastasis and immune modulation, their hierarchical dominance in human OS remains incompletely defined. This network redundancy likely contributes to the limited efficacy of single-agent targeting strategies and underscores the need for combinatorial or systems-level therapeutic approaches.

### 4.2 Tumor–bone microenvironment crosstalk

OS progression is sustained by complex and dynamic interactions between tumor cells and the bone microenvironment, which comprises osteoclasts, osteoblasts, osteocytes, mesenchymal stem cells (MSCs), fibroblastic stromal cells, and vascular components (120–122). Rather than acting as passive bystanders, these bone-resident populations participate in reciprocal signaling circuits that promote tumor expansion, matrix remodeling, angiogenesis, and metastatic competence.

Osteoclasts represent a central effector population within the OS–bone interface. Their differentiation and activation are primarily regulated by macrophage colony-stimulating factor (M-CSF) and RANKL signaling. OS cells actively enhance osteoclastogenesis through production of pro-osteoclastogenic mediators, leading to increased bone matrix degradation. Bone resorption releases matrix-embedded growth factors such as IGF-1 and TGF-β, which in turn stimulate tumor proliferation, invasion, and further secretion of osteolytic mediators, thereby establishing a self-amplifying “vicious cycle” of bone remodeling (**Figure 1**) (123–125). This feed-forward loop links mechanical bone destruction to biochemical tumor stimulation. In preclinical models, inhibition of osteoclast activity reduces tumor-induced bone destruction and metastatic spread, underscoring the relevance of the RANK/RANKL axis in OS progression (116, 125). However, although osteoclast targeting attenuates skeletal damage, its impact on overall survival in clinical settings has been less definitive. In this regard, Endo-Munoz et al. demonstrated that a loss of osteoclasts in the primary tumor site—characterized by a significant downregulation of the osteoclast marker ACP5/TRAP—actually correlates with a higher incidence of lung metastases and a shorter time to metastatic progression. These findings suggest that osteoclasts might paradoxically exert an inhibitory effect on the migration of metastasis-competent cells, implying that OS aggressiveness is not solely dependent on osteoclast-driven resorption but involves a more complex regulatory balance within the bone niche (126).

Osteoblasts and osteocytes contribute to tumor–bone communication by regulating bone formation and skeletal homeostasis. Osteoblast differentiation is controlled by BMP and Wnt/β-catenin pathways and can be altered by tumor-derived signals such as TGF-β and FGF, thereby indirectly promoting remodeling processes that support tumor expansion (26, 127). Osteocytes, as mechanosensitive regulators of bone turnover, coordinate osteoblast–osteoclast coupling and are increasingly considered part of the bone-specific microenvironment that should be recapitulated in biologically relevant OS models (128). Nevertheless, their precise functional contribution in OS remains incompletely characterized, and most available data still derive from bone physiology or model-based systems rather than direct patient-specific functional studies.

MSCs constitute a highly plastic stromal compartment that can be recruited and reprogrammed by OS-derived factors. Upon tumor exposure, MSCs secrete pro-inflammatory and pro-angiogenic mediators including IL-6, IL-8, VEGF, and CCL5, thereby enhancing invasion, angiogenesis, and metastatic dissemination (26, 129). Notably, Baglio et al. demonstrated that OS-derived EVs can educate mesenchymal stem cells toward a pro-inflammatory and pro-metastatic phenotype through TGF-β–mediated induction of IL-6 signaling, thereby promoting STAT3 activation and lung metastasis formation (130). MSC-derived EVs further promote tumor aggressiveness by activating oncogenic signaling pathways, while MSC-mediated IL-6/STAT3 activation contributes to chemoresistance and survival under stress conditions (131). These findings suggest that MSCs function not merely as supportive stromal cells but as active amplifiers of inflammatory and survival circuits. The complexity of the myeloid compartment in this niche is further emphasized by the dual role of TAMs. While generally considered pro-tumorigenic, Gomez-Brouchet et al. showed that high infiltration of CD163+ macrophages and CD8+ lymphocytes in pre-chemotherapy biopsies correlates with improved overall survival. This suggests that the baseline immune composition of the bone niche is a critical determinant of therapeutic response and that TAMs may play a more protective role in OS than in other solid tumors, potentially explaining the limited efficacy of non-selective myeloid-targeting agents (132). However, the stability of MSC reprogramming *in vivo* and the extent to which endogenous MSC populations versus recruited progenitors dominate this process remain areas requiring further investigation, particularly in patient-derived systems.

Fibroblastic stromal cells, including CAF-like populations, are less well resolved in OS than in many epithelial malignancies, but they remain conceptually relevant because matrix remodeling and stromal cytokine production can influence angiogenesis, invasion, immune modulation, and metastatic behavior. By restructuring matrix architecture and modulating stiffness, these stromal populations may also influence mechanotransductive pathways, further integrating biochemical and biomechanical signaling axes. Nonetheless, the specific origin and heterogeneity of CAF-like populations in OS remain insufficiently resolved, limiting precise therapeutic targeting strategies.

Vascular endothelial cells and pericytes are critical regulators of angiogenesis and vascular integrity within the OS microenvironment. Tumor-derived cytokines activate endothelial cells, promoting neovascularization required for tumor growth and facilitating intravasation and hematogenous dissemination. OS-associated vasculature is frequently immature and hyperpermeable, enabling tumor cell trans-endothelial migration (26, 133). Pericytes normally stabilize vascular structures through PDGF-β/PDGFR-β, Ang1, and TGF-β signaling pathways; however, altered pericyte coverage may increase vascular permeability and enhance metastatic spread (26). Growing insight into endothelial–pericyte interactions has supported development of anti-angiogenic strategies targeting VEGF and related pathways in OS (134).

Collectively, reciprocal interactions among tumor cells, bone-resident populations, stromal elements, and vascular compartments generate a dynamic and permissive bone niche that sustains OS growth, angiogenesis, immune modulation, and metastatic progression. Importantly, these compartments converge on shared downstream pathways, including NF-κB, STAT3, PI3K/AKT, MAPK, and Wnt/β-catenin, forming an interconnected signaling network. While the tumor-bone “vicious cycle” model provides a valuable conceptual framework, emerging evidence suggests that OS progression is not solely dependent on osteoclast-driven resorption but instead reflects a broader, multi-compartmental communication system. Therapeutic strategies targeting individual components of this niche may therefore require combinatorial approaches to overcome compensatory signaling redundancy.

### 4.3 Extracellular vesicles

Beyond direct cellular interactions, tumor–microenvironment communication in OS is extensively mediated by EVs, which represent a highly efficient mechanism of protected long-distance signaling (135). Both malignant and stromal cells release EVs containing proteins, lipids, and diverse classes of ncRNAs capable of reprogramming recipient cells and reshaping tumor behavior (136). Unlike soluble mediators, EVs enable coordinated transfer of multiple regulatory molecules, thereby amplifying and stabilizing intercellular communication networks (137).

In OS, EV-associated ncRNAs, including microRNAs, long non-coding RNAs, and circular RNAs, are selectively enriched through endosomal sorting mechanisms involving RNA-binding proteins and vesicular trafficking machinery (135). Once encapsulated within the vesicular membrane, these ncRNAs are protected from degradation in circulation and can modulate gene expression in recipient cells. Functionally, this transfer promotes EMT-like plasticity, cytoskeletal reorganization, metabolic adaptation, and resistance to hostile microenvironmental conditions (135). Activation of downstream pathways such as MAPK/ERK and Wnt/β-catenin, along with upregulation of matrix metalloproteinases, contributes to enhanced invasion and extracellular matrix remodeling (135, 138).

A major consequence of EV-mediated signaling in OS is the establishment of an immunosuppressive microenvironment. Tumor-derived EVs promote M2-like macrophage polarization, expand myeloid-derived suppressor cells (MDSCs), induce regulatory T-cell enrichment, and impair dendritic-cell maturation and antigen presentation (135). These effects collectively suppress cytotoxic T-cell and natural killer cell activity, facilitating immune escape and metastatic progression (139). Importantly, EV-mediated immune modulation operates in parallel with soluble cytokine networks such as IL-6 and IL-8 signaling, suggesting functional redundancy across communication modalities (140).

EVs also contribute to angiogenic regulation. Exosomal ncRNAs modulate endothelial cell proliferation, migration, and tube formation through regulation of VEGF-related signaling and hypoxia-responsive pathways, thereby promoting neovascularization and increased vascular permeability (141). These vascular changes enhance tumor growth and facilitate intravasation into the circulation (135).

Beyond local effects, EVs contribute decisively to pulmonary pre-metastatic niche formation, a critical determinant of OS progression. OS-derived EVs preferentially accumulate in lung tissue, where they reprogram resident fibroblasts and immune cells, remodel extracellular matrix architecture, and increase endothelial permeability (142, 143). Through chemokine and inflammatory signaling pathways, EVs recruit bone marrow-derived suppressive cells and establish an immunologically permissive microenvironment that enhances metastatic seeding efficiency. Specific integrin and glycoprotein profiles expressed on EV membranes may contribute to organotropism, directing vesicle accumulation toward lung tissue and facilitating metastatic colonization (135).

In addition to stromal reprogramming, EV-mediated signaling reinforces tumor-bone interactions by modulating osteoclast differentiation, fibroblast activation, and mesenchymal stromal cell behavior, thereby sustaining feedback loops that support tumor plasticity and invasive growth (135).

Despite strong preclinical evidence supporting these mechanisms, several limitations remain. EV isolation and characterization methods lack full standardization, and many mechanistic studies rely on *in vitro* systems or immunodeficient mouse models that may not fully recapitulate human immune complexity. Furthermore, the heterogeneity of EV populations complicates identification of dominant functional cargo species, and the hierarchical contribution of EV signaling relative to soluble factors in patient tumors remains incompletely defined. Overall, EVs emerge as central mediators of tumor–microenvironment communication in OS, integrating multiple biological processes including immune modulation, angiogenesis, metastasis, and stromal remodeling. This functional centrality provides the mechanistic foundation for the therapeutic strategies discussed in Section 6.3.

### 4.4 Immune communication

In OS, the tumor immune microenvironment (TIME) is shaped by a dynamic and multilayered network of intercellular interactions that collectively determine immune activation, suppression, and therapeutic responsiveness (144). Rather than functioning as isolated cell populations, immune components operate within tightly interconnected feedback loops that reinforce tumor persistence.

TAMs represent a dominant immune population in OS, frequently exhibiting an M2-like polarization state. These cells promote immune evasion through secretion of IL-10 and TGF-β and through expression of immune checkpoints such as PD-L1, thereby fostering T-cell exhaustion and regulatory T-cell (Treg) expansion (19, 139, 145). However, the functional interpretation of TAMs in OS remains controversial. Increasing evidence suggests that macrophages in the bone niche display hybrid M1/M2 phenotypes rather than fitting a binary classification model (139). In line with this context-dependent behavior, Buddingh et al. reported that tumor-infiltrating macrophages were associated with metastasis suppression in high-grade OS, supporting a potential protective and immunomodulatory role of macrophages in specific microenvironmental contexts (146). This plasticity may explain the conflicting clinical data regarding TAM density and prognosis, indicating that spatial organization, functional polarization state, and interaction partners may be more biologically relevant than absolute macrophage abundance (139, 147).

MDSCs further reinforce the immunosuppressive milieu by releasing IL-18 and CXCL12, inhibiting effector T-cell proliferation and skewing immune infiltration toward tolerance (148, 149). Although their interactions with TAMs and DCs are well characterized in experimental systems, the hierarchical dominance of MDSCs within the human OS TIME remains uncertain. Much of the available evidence derives from *in vitro* models that lack the structural and biomechanical complexity of the mineralized bone matrix, potentially oversimplifying immune-cell trafficking and antigen presentation dynamics. The identification of immunoregulatory dendritic-cell subsets (mregDCs) adds an additional layer of immune modulation (150, 151). These cells display tolerogenic features, yet it remains unclear whether they actively initiate immune escape or instead represent adaptive responses to chronic inflammation and tumor-derived conditioning. Resolving this distinction is critical, as therapeutic targeting strategies would differ substantially depending on whether these cells are primary drivers or secondary modulators of disease progression.

Effector T-cells (CD8^+^ and CD4^+^) constitute the principal mediators of anti-tumor immunity, but their function in OS is frequently impaired. Comprehensive immuno-transcriptomic analyses demonstrated that OS exhibits relatively high immune-cell infiltration compared with several other pediatric solid tumors, with increased CD8+ T-cell signatures and intratumoral TCR clonality correlating with improved patient survival. However, these tumors also displayed elevated expression of immunosuppressive mediators, including TGFB1 and CSF1R, highlighting the coexistence of immune activation and inhibitory signaling within the OS microenvironment (152). Cytokine-mediated feedback loops involving IL-10, IL-35, and TGF-β, together with checkpoint signaling through CTLA-4 and TIGIT, contribute to progressive T-cell dysfunction and exhaustion (153–155). Importantly, many studies rely on bulk transcriptomic or immunohistochemical analyses that may obscure functional heterogeneity. Distinguishing transiently dysfunctional T-cells from terminally exhausted subsets requires spatially resolved and single-cell approaches, which remain underutilized in OS research. This immune communication network is further reinforced by tumor-derived exosomes and soluble mediators, which promote M2 polarization and impair DC maturation (144, 147). Thus, immune suppression in OS is not the consequence of a single dominant pathway but rather the result of converging cellular and vesicular signaling axes.

Overall, while the cellular architecture of the OS TIME is increasingly well defined, critical gaps remain in understanding the triggers of macrophage plasticity, the spatial dynamics of immune exhaustion, and the relative hierarchy of suppressive cell populations. Addressing these unresolved questions will be essential to move beyond descriptive immune profiling toward rational and personalized immunotherapeutic strategies.

### 4.5 Direct cell–cell communication in osteosarcoma

Beyond soluble mediators and EVs, OS cells engage in direct physical communication that coordinates proliferation, invasion, and stromal integration. Two principal mechanisms have emerged as particularly relevant: gap junctions (GJs), predominantly mediated by Connexin43 (Cx43), and tunneling nanotubes (TnTs).

GJs enable direct cytoplasmic exchange of ions and small signaling molecules between adjacent cells. In OS, Cx43 has traditionally been described as a tumor suppressor because its expression can slow G1/S cell-cycle progression through p27 upregulation (156). However, this characterization appears overly simplistic. While elevated Cx43 expression may restrain proliferation, GJ-mediated coupling with osteoblasts can facilitate TGF-β-driven EMT-like programs and enhance invasive behavior. This apparent duality suggests that Cx43-dependent communication may shift tumor-cell priorities from proliferation toward dissemination depending on microenvironmental cues. The molecular determinants that govern this functional switch remain undefined, representing a significant conceptual gap in OS biology (156).

TnTs represent a complementary communication system. These actin-based membranous bridges connect distant cells and permit transfer of complex cargo, including mitochondria and regulatory RNAs such as miR-19a (157). Through organelle exchange and metabolic support, TnTs may enhance survival under stress conditions and potentially contribute to chemoresistance. However, the study of TnTs in OS faces important methodological limitations. Most observations derive from two-dimensional *in vitro* systems, where these delicate structures are more easily visualized (158). Their structural stability and functional relevance within the dense, mineralized extracellular matrix of a three-dimensional tumor remain insufficiently validated. Moreover, the absence of specific pharmacological inhibitors limits the ability to establish causality, leaving much of the current evidence associative rather than mechanistically definitive.

Together, GJs and TnTs form a hierarchical network of direct intercellular communication within the OS microenvironment. GJs mediate rapid, localized synchronization of adjacent cells via exchange of small signaling molecules, whereas TnTs enable long-distance transfer of macromolecules and organelles capable of reprogramming recipient-cell metabolism and behavior. This dual communication architecture likely contributes to intratumoral heterogeneity, coordinated tumor-stroma adaptation, and metastatic competence. Nevertheless, translating these findings into therapeutic strategies requires deeper mechanistic clarification, particularly regarding the contextual regulation of Cx43 function and the *in vivo* persistence of TnTs within bone tissue. Without such validation, targeting these pathways risks oversimplifying a highly dynamic and spatially constrained communication landscape.

## 5 Functional consequences of intercellular communication

The convergence of soluble mediators, EVs, and direct physical contacts establishes a multilayered communication network that ultimately dictates the functional trajectory of OS. Rather than acting through isolated pathways, these communication axes integrate to drive three major biological outcomes: local bone destruction and tumor expansion, metastatic dissemination with immune escape, and adaptive resistance to therapy.

At the primary tumor site, the communication mechanisms described in Sections 4.1 and 4.2 converge to reshape the bone microenvironment by coupling inflammatory cytokine signaling with osteoclast-dependent bone remodeling. These integrated communication circuits establish the well-characterized “vicious cycle” of tumor-induced bone destruction, which sustains tumor growth, angiogenesis, and local invasion (84, 85).

As discussed in Sections 4.3 and 4.4, EV-mediated signaling cooperates with immune-cell communication to extend tumor influence beyond the primary site, promoting pulmonary niche conditioning, immune suppression, and metastatic colonization (135). These processes demonstrate that metastasis in OS is not solely dependent on tumor-cell intrinsic properties but is critically conditioned by systemic communication programs.

Direct cell-cell communication further reinforces these processes by coordinating tumor-cell behavior through gap junctions and tunneling nanotubes, which promote metabolic adaptation, stress tolerance, and collective survival (156, 157). Collectively, these communication modalities converge on signaling pathways such as STAT3, PI3K/AKT, and Wnt/β-catenin, ultimately supporting therapy resistance and tumor persistence.

Collectively, intercellular communication in OS functions as a dynamic coordination system that links bone destruction, immune evasion, metastatic competence, and therapeutic resistance. Understanding the hierarchy and temporal activation of these interconnected pathways will be essential to identify which communication nodes represent true therapeutic vulnerabilities rather than secondary epiphenomena.

## 6 Targeting communication networks as therapeutic strategy in osteosarcoma

**Figure 2:**
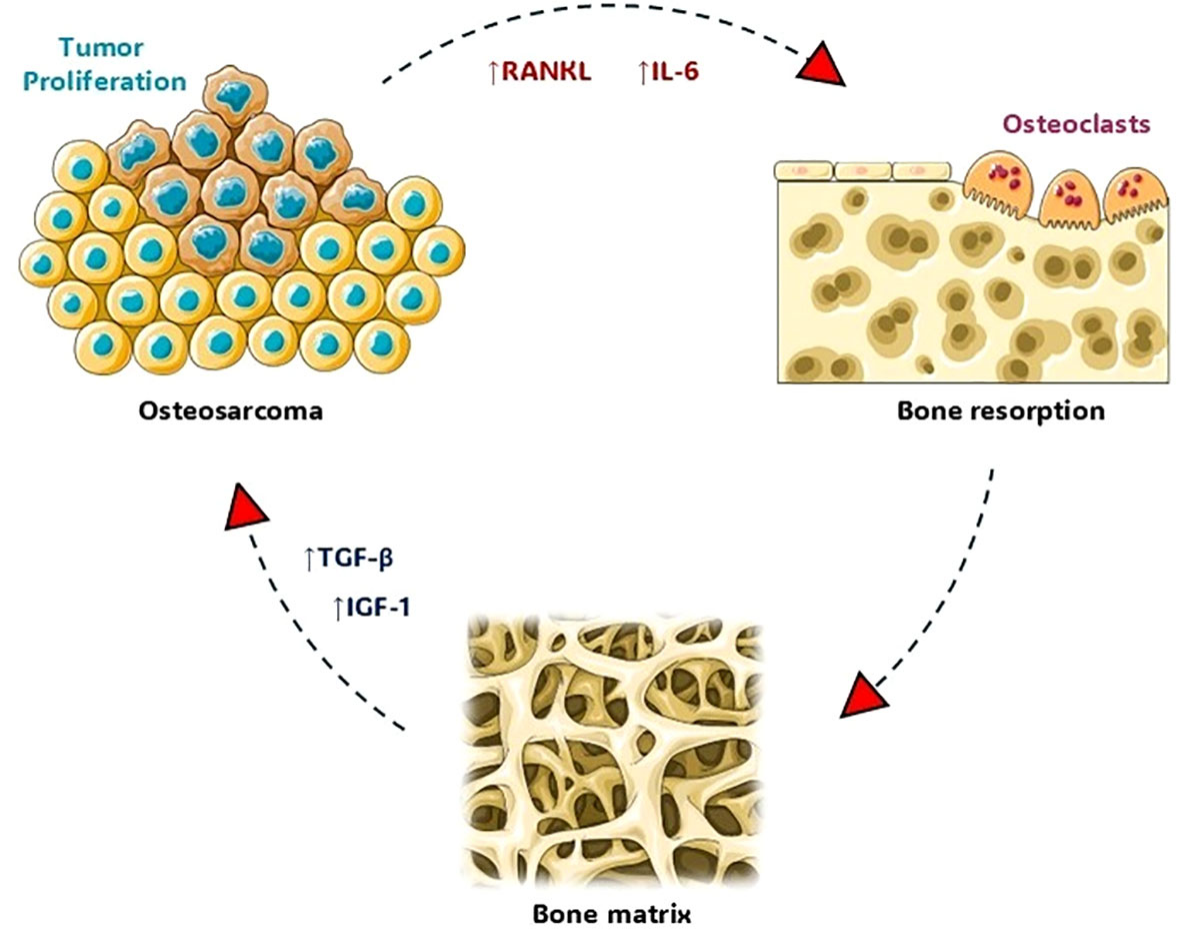
Multi-level therapeutic strategies targeting the osteosarcoma microenvironment. The image illustrates three main intervention axes: **(A)** Targeting Soluble Mediators, focusing on the inhibition of cytokines and growth factors; **(B)** Targeting EV-Mediated Communication, involving the suppression of oncogenic ncRNA cargo, restoration of tumor-suppressive ncRNAs, and inhibition of vesicle biogenesis; and **(C)** Therapeutic Modulation of Immune Cell Crosstalk, aimed at macrophage reprogramming, dendritic cell activation, T-Cell-based immunotherapy, and adoptive T-Cell therapy. These combined strategies aim to attenuate tumor growth, metastasis, angiogenesis, and osteolysis.

Targeting intercellular communication networks has emerged as a complementary therapeutic paradigm in OS, aiming to disrupt the signaling circuits that connect tumor cells with immune, stromal, and bone microenvironment components. Rather than focusing exclusively on tumor-intrinsic oncogenic drivers, these approaches attempt to dismantle the extrinsic communication hubs that sustain proliferation, immune evasion, osteolysis, and metastatic dissemination. The principal communication-oriented strategies discussed in this review are summarized in **Figure 2**.

### 6.1 Clinical evidence and current therapeutic landscape in osteosarcoma

Despite extensive preclinical evidence supporting communication-targeted strategies in OS, clinical translation remains limited and only a small number of approaches have demonstrated reproducible activity in patients. A summary of completed, ongoing, and planned clinical trials evaluating these therapeutic strategies is provided in **Table 2**. Among communication-oriented interventions, anti-angiogenic multi-kinase inhibitors targeting VEGF-dependent tumor-microenvironment signaling represent the most clinically validated strategy in relapsed or refractory disease. Randomized phase II trials, including the SARC024 and REGOBONE studies, demonstrated that regorafenib significantly improved progression-free survival compared with placebo, supporting angiogenic signaling as a relevant therapeutic vulnerability, although responses are predominantly disease-stabilizing rather than curative (176, 177). Other multi-kinase inhibitors, such as sorafenib, have shown similar disease-control activity in non-randomized or early-phase studies, further confirming the role of microenvironmental signaling pathways in OS progression (178).

**Table 2:** Summary of completed and ongoing clinical trials evaluating communication-related therapeutic strategies in osteosarcoma.

Trial/study name	Phase	Drug/intervention	Status	Population & key details/results
REGOBONE (French Sarcoma Group) (159)	2	Regorafenib vs. Placebo	Completed	Met primary endpoint: PFS-8wk was 65% for regorafenib vs. 0% for placebo.
SARC038 (160)	2	Regorafenib + Nivolumab	Ongoing	Active study evaluating the combination of TKI and anti-PD-1; results not yet reported.
REGOSTA (161)	2	Regorafenib vs. Placebo	Completed	Evaluated regorafenib as maintenance therapy after first-line treatment.
CABONE (162)	2	Cabozantinib	Completed	6-month PFS was 33% (95% CI 20–50) in the osteosarcoma/Ewing sarcoma cohort.
Cabozantinib + Chemo (163)	2/3	Cabozantinib + Chemotherapy	Planned	Designed for newly diagnosed osteosarcoma cases.
Sorafenib + Everolimus (ISG) (164)	2	Sorafenib + Everolimus	Completed	Italian Sarcoma Group trial. PFS-6mo was 45% (95% CI 28–61); showed no overall survival (OS) benefit compared to historical controls.
Pazopanib (Lung Metastases) (165)	2	Pazopanib	Completed	Demonstrated disease stabilization in lung-metastatic osteosarcoma; tolerability profile confirmed.
OAIE/PKUPH-sarcoma 11 (166)	3	Apatinib + IE vs. IE alone	Completed	Apatinib + Ifosfamide/Etoposide (IE) prolonged mPFS to 5.5 months (95% CI 3.9–6.7) compared to 3.4 months for IE alone (95% CI 1.4–4.6).
APFAO (Peking University) (167)	2	Apatinib + Camrelizumab	Completed	Combination of TKI and anti-PD-1 checkpoint inhibitor achieved a PFS-6mo of 54.3% (95% CI 37.6–68.3).
Lenvatinib ± Everolimus (EVERLA) (168)	2	Lenvatinib ± Everolimus	Planned	Investigating combination strategies in bone sarcomas.
Lenvatinib + IE (169)	2	Lenvatinib + IE vs. IE	Completed	Evaluated in relapsed osteosarcoma; full efficacy results are pending formal publication.
PEMBROSARC (170)	2	Pembrolizumab + Cyclophosphamide	Completed	Pembrolizumab combined with metronomic cyclophosphamide. Objective Response Rate (ORR) at 6 months in the OS cohort was 0% (95% CI 0–0).
PIVOT IO 020 (171)	1/2	Bempegaldesleukin + Nivolumab	Completed	IL-2 agonist combined with anti-PD-1; reported 0 objective responses within the osteosarcoma cohort.
Autologous NK ± rhIL-15 (172)	1	Autologous NK cells ± rhIL-15	Completed	Dose-escalation study demonstrating the technical feasibility of NK cell manufacturing and therapeutic infusion.
SARCOME13 (173)	2	Mifamurtide + Post-op Chemo	Ongoing	Evaluating the addition of mifamurtide to standard postoperative chemotherapy versus chemotherapy alone.
Denosumab (Recurrent/Refractory) (174)	2	Denosumab	Completed	Assessed oncologic activity in relapsed osteosarcoma; revealed a limited efficacy signal.
HS-20093 vs. Gem/Doc (175)	3	HS-20093 vs. Gemcitabine + Docetaxel	Ongoing	First Phase 3 B7-H3-targeted antibody-drug conjugate (ADC) clinical trial in osteosarcoma.

Therapeutic modulation of tumor-immune communication has also reached clinical evaluation. Macrophage activation with mifamurtide, investigated in randomized studies in combination with chemotherapy, represents one of the few immunomodulatory strategies incorporated into clinical practice in selected settings, although the magnitude of survival benefit remains debated and its adoption varies across regulatory contexts (179). In contrast, immune checkpoint blockade targeting PD-1/PD-L1 signaling has shown limited efficacy as monotherapy in OS, highlighting the complexity and redundancy of immune suppression within the tumor microenvironment. Emerging clinical studies are currently exploring combinatorial approaches integrating anti-angiogenic agents with immune checkpoint inhibition or chemotherapy, aiming to simultaneously modulate multiple communication networks (180).

Collectively, available clinical evidence indicates that disruption of tumor–microenvironment communication can achieve measurable biological and clinical effects, yet durable survival benefits remain modest. These observations underscore the persistent gap between mechanistic insights into intercellular signaling networks and effective therapeutic translation, supporting the need for biomarker-driven patient stratification and rational combination strategies targeting multiple interconnected communication pathways.

### 6.2 Targeting soluble mediators in osteosarcoma

#### 6.2.1 IL-6/STAT3 axis

Given its central role in OS–microenvironment crosstalk, the IL-6/IL-6R/JAK/STAT3 axis represents one of the most mechanistically validated communication targets (see Sections 3.2 and 4.1.1 for detailed signaling and biological effects). Preclinical studies demonstrate that IL-6R blockade with tocilizumab suppresses STAT3 phosphorylation, reduces invasive potential, reverses EMT-associated phenotypes, and restores sensitivity to tyrosine kinase inhibitors (21, 90, 181). Despite the strong biological rationale, clinical validation remains limited. Cytokine redundancy within the OS microenvironment raises concerns that IL-6 inhibition alone may not produce durable responses. Consequently, IL-6 blockade is unlikely to be effective as monotherapy and appears more rational within combination regimens targeting complementary inflammatory or survival pathways.

#### 6.2.2 TGF-β signaling

TGF-β represents another central regulator of OS-microenvironment communication as already described in Section 4.1.2. Multiple therapeutic approaches have been developed to disrupt this axis, including neutralizing antibodies, ligand traps, antisense oligonucleotides, TβRI/ALK5 kinase inhibitors, vaccines, miRNA- and siRNA-based strategies, and SMAD7 overexpression (94). Preclinical data consistently demonstrate that small-molecule inhibitors such as SB-431542, LY2109761, RepSox, and Vactosertib suppress proliferation, inhibit EMT-associated transcriptional programs, reduce lung metastasis, and partially restore anti-tumor immune infiltration (182–185). However, TGF-β signaling is pleiotropic and context-dependent, exerting both tumor-suppressive and tumor-promoting effects depending on disease stage and cellular environment (186–188). This biological duality complicates therapeutic targeting and raises the risk of unintended consequences. Although early clinical experiences indicate acceptable safety profiles, efficacy as monotherapy has been modest. These observations suggest that TGF-β inhibition may require integration with immunotherapy or other microenvironment-targeted strategies to achieve meaningful clinical impact (99).

#### 6.2.3 VEGF axis

Among communication-oriented strategies, angiogenic targeting represents the most clinically validated approach in relapsed or metastatic OS. Multi-kinase inhibitors targeting VEGFR signaling, including regorafenib, cabozantinib, sorafenib, and pazopanib, have demonstrated reproducible activity in phase II trials, primarily prolonging progression-free survival rather than inducing durable tumor regressions (107). Regorafenib significantly improved progression-free survival in randomized phase II studies and is currently recommended for refractory disease (176, 177). Cabozantinib showed meaningful activity, partly through dual inhibition of VEGFR2 and MET, thereby influencing both tumor vascularization and bone microenvironment dynamics (107). Sorafenib, alone or combined with mTOR inhibitors, enhanced suppression of tumor growth and metastatic potential (189). Additional VEGFR-targeting agents such as apatinib and lenvatinib further confirmed the role of angiogenic signaling in OS progression, showing disease stabilization and modulation of EMT and STAT3-dependent pathways (107). Nevertheless, overall survival benefits remain limited, underscoring that anti-angiogenic therapy predominantly exerts disease-stabilizing rather than curative effects and highlighting the need for biomarker-driven patient selection and rational combinations.

#### 6.2.4 CXCL12/CXCR4 axis

As already discussed in Section 4.1.4., the CXCL12/CXCR4 axis represents an important determinant of poor clinical outcome (190). Pharmacological inhibition with AMD3100 or anti-CXCR4 antibodies reduces migration, invasion, and pulmonary metastases *in vivo*. Combination approaches, including CXCR4 blockade with NK-cell therapy, further enhance anti-metastatic efficacy by limiting dissemination and promoting immune-mediated clearance. However, clinical translation remains preliminary (190, 191).

#### 6.2.5 RANK/RANKL pathway

The RANK/RANKL axis represents a central mediator of tumor-induced osteolysis. Targeting this pathway through osteoprotegerin (OPG), soluble RANK-Fc, RNA interference against RANKL, or monoclonal antibodies such as denosumab reduces osteoclast activity and limits tumor growth in preclinical models (192). Clinically, denosumab provides sustained inhibition of bone resorption with favorable safety and convenient administration (193). While its efficacy is well established in other bone-related malignancies, its precise impact on long-term oncologic outcomes in OS remains to be clarified. Emerging agents such as the camelid-derived antibody ALX-0141 demonstrate potent inhibition of bone turnover markers and good tolerability in early studies (192).

#### 6.2.6 CCL2/CCR2 axis

The CCL2–CCR2 pathway exemplifies the challenges of targeting microenvironmental signaling. While CCR2 inhibition modulates cytokine secretion, hypoxia-associated markers, immune checkpoint expression, and angiogenic mediators in experimental systems, these biological effects do not consistently translate into significant tumor growth suppression *in vivo* (194, 195). Compensatory activation of alternative pathways and hypoxia-driven adaptations likely undermine single-agent efficacy (195).

Collectively, therapies targeting OS–microenvironment communication networks have generated compelling preclinical evidence but limited transformative clinical benefit. The modest activity observed with single-agent approaches reflects the intrinsic redundancy, spatial heterogeneity, and adaptive plasticity of the OS microenvironment. Future therapeutic advances will likely depend on biomarker-driven patient stratification, temporal targeting of dominant communication nodes, and rational combination regimens capable of simultaneously disrupting multiple interconnected signaling pathways. Rather than targeting isolated mediators, effective strategies will need to dismantle the broader communication architecture that sustains tumor survival and progression.

### 6.3 Therapeutic reprogramming and targeting of EV-mediated communication

Given the central role of EV-associated ncRNAs in orchestrating OS progression, therapeutic strategies targeting EV-mediated communication follow two complementary directions: suppression of oncogenic vesicular signaling and reprogramming of EV biology for therapeutic delivery.

#### 6.3.1 Targeting EV cargo: rebalancing the ncRNA network

According to what has been discussed in Section 4.3, the direct targeting of pathogenic EV cargo represents a rational downstream therapeutic strategy. Antisense oligonucleotides and siRNA-based approaches directed against oncogenic lncRNAs such as CASC15 and EWSAT1 inhibit migration, angiogenesis, and tumor growth in preclinical models (20, 196). Similarly, anti-miR strategies targeting miR-21 and restoration of tumor-suppressive lncRNAs such as GAS5 demonstrate the feasibility of shifting the ncRNA balance toward anti-tumor signaling. Conversely, therapeutic replacement strategies aim to restore tumor-suppressive miRNAs. Delivery of miR-206 via BMSC-derived exosomes and administration of miR-101 to inhibit pulmonary metastasis provide proof-of-concept evidence that ncRNA supplementation can attenuate OS progression (20, 197). While biologically compelling, these approaches remain largely confined to preclinical settings, and challenges related to delivery efficiency, off-target effects, and durability of response remain unresolved.

#### 6.3.2 Targeting EV biogenesis and uptake: upstream interference

Rather than targeting individual EV cargo molecules (Section 6.3.1), alternative strategies aim to interfere upstream with EV production, release, or cellular uptake, thereby limiting the communication networks described in Section 4.3. Inhibition of neutral sphingomyelinase with GW4869 can reduce exosome release in experimental systems, although OS-specific validation remains limited and this approach should be considered a mechanistic proof-of-concept rather than an established OS-directed strategy (198, 199). Additional strategies include interference with tetraspanins or integrin-mediated interactions, which may impair EV docking and internalization.

Beyond quantitative inhibition, qualitative modulation of EV content has also been explored. For instance, activation of cannabinoid receptor signaling using WIN has been shown to alter EV cargo composition and attenuate their pro-migratory signaling properties (200, 201). These findings suggest that reprogramming EV output may be more effective than global suppression, although specificity and systemic consequences remain unresolved.

However, given the heterogeneity of EV populations and their role in physiological intercellular communication, broad inhibition strategies raise important concerns regarding safety and potential off-target systemic effects.

#### 6.3.3 Engineering EVs as therapeutic platforms

In parallel with inhibition strategies, EVs can be exploited as naturally biocompatible nanocarriers for therapeutic delivery, leveraging their intrinsic stability, low immunogenicity, and tissue tropism.

Engineering approaches include genetic and chemical modifications to enhance targeting efficiency and pharmacokinetics. These include ligand display systems such as LAMP2B or PDGFR scaffolds, RGD-mediated targeting motifs, click chemistry functionalization, and PEGylation strategies to improve circulation stability and tumor specificity (202, 203). EV-based drug delivery systems have demonstrated promising results in OS models. For example, exosome-encapsulated doxorubicin (Exo-Dox) enhances anti-tumor efficacy while reducing systemic toxicity compared to free drug administration (204). In addition, hybrid EV–synthetic nanocarriers, generated via liposomal fusion or polymer integration, further expand loading capacity and enable controlled drug release profiles (135). Despite their potential, several translational barriers remain, including lack of standardization in EV isolation, scalability of production, variability in cargo loading efficiency, batch-to-batch reproducibility, and regulatory classification challenges. Furthermore, the biological heterogeneity of EV populations complicates quality control and predictive therapeutic performance.

Collectively, therapeutic strategies targeting EVs translate the biological framework described in Section 4.3 into two main directions: inhibition of pathological EV-mediated communication and exploitation of EVs as customizable drug delivery systems. Although conceptually promising, most approaches remain at preclinical or early translational stages. Future progress will require improved understanding of EV functional hierarchy in OS, standardization of manufacturing pipelines, and careful evaluation of systemic effects to enable safe clinical translation.

### 6.4 Therapeutic modulation of immune cell crosstalk

Given the central role of TAMs in orchestrating immune communication within the OS microenvironment, therapeutic strategies increasingly aim to reprogram macrophage polarization and disrupt pro-tumoral feedback loops rather than simply deplete immune populations (205).

#### 6.4.1 Macrophage reprogramming strategies

Based on the functional plasticity of TAMs and their context-dependent role in OS, as discussed in Section 4.4, therapeutic strategies aimed at reprogramming or activating macrophage populations rather than simply depleting them may represent a rational approach to restore anti-tumor immune activity. Several pharmacological and biological approaches have been developed to exploit this principle. Among differentiation- and polarization-modulating agents, all-trans retinoic acid (ATRA) promotes pro-inflammatory macrophage activation by enhancing NLRP3 inflammasome priming through NF-κB and ERK signaling pathways in human macrophages (206). In OS preclinical models, ATRA inhibits M2-like polarization and reduces tumor initiation, cancer stemness, and metastatic potential (207), and its clinical relevance is supported by its use in combination with interferon-α in patients with resistant metastatic disease (208). Similarly, natural compounds such as asiaticoside and esculetin attenuate M2-associated signaling programs, thereby weakening tumor-supportive macrophage functions (209, 210). Additional approaches, including photothermal therapy combined with nanomaterials, further promote macrophage reprogramming toward inflammatory phenotypes while limiting M2 polarization (211). Clinically, mifamurtide remains the most established macrophage-modulating agent in OS, influencing pAKT/STAT3 signaling and RANK-related pathways to reinforce pro-inflammatory immune communication (204, 212, 213). Additional strategies include blockade of the CSF1/CSF1R axis (e.g., pexidartinib), which interferes with macrophage recruitment and survival, and activation of TLR7/8 signaling (resiquimod), which enhances TAM reprogramming and DC engagement (214–216). Targeting communication interfaces such as CD47/SIRPα or stimulating CD40 further aims to restore phagocytosis and amplify macrophage–T-cell crosstalk (204). Intracellular hubs including PI3Kγ and ERK5/MAPK represent additional nodes capable of reshaping TAM-driven networks (217, 218). Despite strong mechanistic rationale, most macrophage-targeted strategies remain preclinical or early clinical. Moreover, macrophage plasticity and spatial heterogeneity continue to represent major challenges for predicting therapeutic efficacy, suggesting that context-dependent responses may significantly influence clinical outcomes.

#### 6.4.2 Dendritic cell-mediated immune communication

DCs are potent APCs that prime T-cell responses against tumors and regulate antigen presentation within the OS microenvironment (219). DC-based vaccines represent a promising strategy for OS immunotherapy, aiming to enhance anti-tumor immunity by delivering OS-associated antigens, such as tumor lysates or synthetic peptides, to the immune system (220). While clinical trials using autologous DC vaccines pulsed with tumor lysates reported prolonged progression-free survival in subsets of patients, overall response rates remained low (220). To overcome these limitations, novel findings highlight the superior efficacy of CD103^+^ conventional type 1 DC (cDC1) vaccines. Unlike traditional monocyte-derived vaccines, CD103^+^ cDC1s specifically enhance systemic cytotoxic T-cell activation and promote deep infiltration of CD8^+^ T-cells into both primary tumors and established lung metastases (221). Furthermore, combination strategies are being explored to boost effectiveness: integrating DC vaccines with checkpoint inhibitors (e.g., anti-PD-1 or anti-CTLA-4) has shown synergistic tumor rejection in OS models (221), while combinations with oncolytic viruses or agents like capsaicin can further amplify DC-mediated T-cell priming and phagocytosis (222). Despite these advancements, the dual role of DCs, which can turn tolerogenic without appropriate activation signals, underscores the need for precise modulation and optimized platforms to achieve durable therapeutic outcomes (223).

#### 6.4.3 Immune checkpoint blockade

Immune checkpoint inhibitors (ICIs) target inhibitory signaling axes that suppress cytotoxic T-cell function. In OS, PD-L1 expression within tumor or immune compartments and PD-1-related immune suppression provide a biological rationale for checkpoint blockade, but clinical outcomes have been modest (19, 148, 186).

Single-agent PD-L1 blockade with atezolizumab failed to show meaningful responses in early-phase trials (224). Combination regimens such as nivolumab plus ipilimumab or durvalumab plus tremelimumab produced limited efficacy in small cohorts (225). Likewise, nivolumab combined with the IL-2 agonist bempegaldesleukin yielded no objective responses despite relatively higher tumor mutation burden compared to other sarcomas (226–228). Similarly, single-agent PD-1 blockade with pembrolizumab demonstrated minimal activity in advanced OS in the PEMBROSARC study (170). Importantly, PD-L1 expression and tumor mutation burden have not consistently predicted therapeutic response in OS (226), suggesting that immune suppression in this disease extends beyond single checkpoint pathways and should be interpreted through broader, multi-parameter immune profiling rather than single-marker assessment.

Combinatorial approaches integrating immune checkpoint blockade with anti-angiogenic targeting remain under active investigation. Ongoing studies such as SARC038 evaluate the combination of regorafenib and nivolumab in refractory or recurrent OS, reflecting the rationale for simultaneous angiogenic and immune modulation (180). In contrast, pembrolizumab combined with cyclophosphamide showed limited benefit (170), reinforcing the need for rational rather than empirical combinations.

#### 6.4.4 Adoptive and cell-based immunotherapies

Adoptive T-cell strategies, including cytotoxic T lymphocyte transfer, γδ T-cell therapy, and gene-engineered tumor-specific T-cells, aim to directly enhance tumor recognition and cytolytic activity (13). Chimeric antigen receptor (CAR)-T-cells targeting OS-associated antigens such as HER2, GD2, and B7-H3 promote antigen-specific immune synapse formation and tumor cell killing (229–231). However, persistence, trafficking efficiency, and immunosuppressive niche conditioning remain major obstacles. Modulation of the microenvironment through CSF1R inhibition has been shown to reduce TAM and Treg populations while increasing CD8^+^ T-cell infiltration, illustrating the interconnected regulation of innate and adaptive immune signaling (204, 214).

#### 6.4.5 Emerging immune checkpoints and humoral immunity

Additional immune checkpoints, including HVEM/CD270 and B7-H3/CD276, have been identified in OS lesions and correlate with T-cell infiltration patterns, representing potential new therapeutic targets (170). Dual checkpoint blockade combined with radiotherapy or metabolic interventions such as L-arginine supplementation enhances CD8^+^ T-cell recruitment and effector function, highlighting the importance of coordinated immune modulation (232).

B cells and humoral immunity also contribute to intercellular communication. Increased infiltration of activated and memory B cells correlates with improved survival (13). B cell–derived antibodies mediate antibody-dependent cellular cytotoxicity, complement activation, and modulation of tumor receptor signaling. However, certain antibody responses may paradoxically dampen immune-mediated tumor clearance, underscoring the complexity of humoral regulation (13).

#### 6.4.6 Natural killer cells

Natural killer (NK) cells participate in immune communication by recognizing tumor cells with reduced MHC class I expression and mediating direct cytotoxicity (13). Therapeutic strategies including adoptive NK cell transfer, IL-15–based activation protocols, and CAR-engineered NK cells aim to restore innate immune surveillance (233). While promising, these approaches face challenges related to *in vivo* persistence and tumor microenvironment suppression.

Overall, immune-targeted therapies in OS have demonstrated limited efficacy when applied as single agents. The restricted activity of checkpoint blockade highlights the redundancy and multilayered nature of immune suppression within the OS microenvironment. These data collectively suggest that effective immunotherapy in OS will likely require combinatorial strategies capable of simultaneously modulating macrophage polarization, angiogenic signaling, antigen presentation, and T-cell cytotoxic communication. Therapeutic success will depend not on isolated pathway inhibition but on coordinated rewiring of innate and adaptive immune signaling networks within the tumor ecosystem.

To provide an integrated overview of the communication networks discussed throughout this review, the major pathways are functionally categorized according to their predominant biological roles, their relative therapeutic relevance, and their current level of translational maturity (**Table 3**). Although many signaling axes contribute simultaneously to multiple hallmarks of OS progression, only a limited number have progressed beyond preclinical investigation toward clinical validation, highlighting the need to prioritize the most therapeutically actionable communication pathways and to develop rational combination strategies targeting complementary signaling networks.

**Table 3:** Functional classification of the major communication pathways involved in osteosarcoma progression according to their principal biological roles and current translational status. Major communication pathways are categorized based on their contribution to metastasis, immune evasion, angiogenesis, chemoresistance, and bone destruction, together with an appraisal of the current level of clinical translation.

Communication pathway/network	Metastasis	Immune evasion	Angiogenesis	Chemoresistance	Bone destruction	Current translational status	Therapeutic relevance
IL-6/STAT3 signaling	++	+	+	++	+	Predominantly preclinical. Strong mechanistic evidence supporting metastatic dissemination, inflammatory signaling, and therapy resistance; IL-6R blockade has not yet achieved clinical validation in OS (21, 42, 90–93, 181).	High
TGF-β signaling	++	++	+	++	++	Predominantly preclinical. Extensive experimental evidence links TGF-β to invasion, immune suppression, osteolysis, and treatment adaptation, although its pleiotropic biology complicates therapeutic targeting (94–97, 123, 182, 184–186).	High
VEGF/VEGFR signaling	++	+	++	+	+	Clinically validated. Anti-angiogenic multi-kinase inhibitors (e.g., regorafenib, cabozantinib, sorafenib) have demonstrated clinical activity, mainly resulting in disease stabilization rather than durable responses (105–107, 176, 177, 234).	High
CXCL12/CXCR4 signaling	++	+	–	+	–	Early clinical/predominantly preclinical. Strong evidence supports its role in pulmonary metastasis, while clinical translation remains limited (84, 85, 101, 190, 191).	Moderate
CCL2/CCR2 signaling	++	++	–	–	–	Preclinical. Experimental studies support its role in macrophage recruitment and pre-metastatic niche formation, but no clinically validated therapeutic strategy is currently available (117, 118, 194, 195).	Low
IL-8/CXCR1/2 signaling	++	+	+	–	–	Preclinical. Promotes inflammatory crosstalk, angiogenesis, and metastatic behavior, although therapeutic inhibition remains experimental (119).	Low
RANK/RANKL signaling	+	–	–	–	++	Limited clinical evidence. Strong biological rationale for targeting bone remodeling, but the oncologic benefit of anti-RANKL therapies in OS remains uncertain (116, 124–126, 192, 235).	Moderate
Extracellular vesicle (EV)-mediated communication	++	++	+	++	+	Preclinical. Extensive evidence demonstrates roles in stromal reprogramming, immune modulation, metastatic niche formation, and drug resistance, although translation is hindered by EV heterogeneity and lack of standardized targeting approaches (20, 24, 100, 135, 141, 142, 196, 197).	Moderate
Immune-cell communication networks (TAMs, MDSCs, T cells, DCs)	+	++	–	+	–	Limited clinical validation. Immunomodulatory strategies have entered clinical evaluation, but single-agent checkpoint blockade has shown limited efficacy in OS (19, 132, 144–147, 205, 207, 209, 212–215).	Moderate
Gap junctions (Connexin43)	+	–	–	+	–	Preclinical. Experimental evidence suggests context-dependent effects on invasion and treatment adaptation, but no clinically applicable targeting strategy is available (25, 156).	Exploratory
Tunneling nanotubes (TNTs)	+	–	–	+	–	Preclinical. Emerging evidence indicates roles in metabolic cooperation and stress adaptation, although *in vivo* validation remains limited (157, 158).	Exploratory

++, strong and consistently supported evidence in osteosarcoma; +, moderate, context-dependent, or limited evidence; −, no substantial evidence or minor contribution reported to date.

## 7 Emerging technologies and future perspectives

Emerging high-resolution molecular technologies are redefining the biological landscape of OS by enabling precise dissection of tumor heterogeneity and intercellular communication networks (236). These approaches are shifting OS research from bulk descriptive models toward spatially and temporally resolved systems biology.

Single-cell RNA sequencing (scRNA-seq) has revealed the remarkable cellular diversity within OS, identifying malignant subpopulations spanning proliferative, mesenchymal stem cell–like, and osteoblastic lineage states, as well as distinct immune and stromal compartments (237, 238). Beyond simple cell identification, these datasets reconstruct lineage trajectories and plastic transitions, uncovering immune subsets enriched in metastatic lesions, including pro-inflammatory macrophage populations and myeloid phenotypes associated with T-cell exhaustion (237–239). Such findings provide mechanistic insight into immune escape and suggest candidate biomarkers for therapeutic stratification.

Spatial transcriptomics adds a critical architectural dimension by preserving tumor–stroma topology. Integrative analyses combining scRNA-seq and spatial profiling have identified discrete ecological niches, such as fibroblast-dense regions adjacent to necrotic zones, associated with chemoresistance and altered communication programs that would remain undetected in dissociative analyses (236, 240). These spatial gradients help explain immune exclusion, angiogenic heterogeneity, and localized signaling dominance within the tumor ecosystem.

Liquid biopsy technologies, including circulating tumor DNA (ctDNA) and EV analysis, offer minimally invasive tools for longitudinal disease monitoring (241, 242). Multi-omic platforms integrating genomic, proteomic, and metabolomic data have detected early molecular alterations preceding radiographic metastasis, supporting adaptive treatment strategies. Although low tumor DNA shedding and bone-derived cfDNA interference limit sensitivity in OS, advances in ultra-deep sequencing and fragmentomic analyses are progressively enhancing detection performance (243).

At a systems level, integrative computational frameworks such as Multi-Omics Factor Analysis (MOFA) and Similarity Network Fusion (SNF) are enabling identification of latent regulatory circuits across multi-layered datasets (244, 245). When combined with single-cell multi-omics, simultaneously profiling genomic, transcriptomic, and epigenomic features, these approaches allow dynamic tracking of subclonal evolution, immune escape, and therapy resistance (246).

Collectively, the convergence of single-cell, spatial, and liquid biopsy technologies heralds a transition from static tumor profiling toward dynamic mapping of communication networks. The major translational challenge will be transforming high-dimensional datasets into clinically actionable biomarkers and therapeutic decision algorithms.

## 8 Limitations of the current evidence

Several limitations should be considered when interpreting OS through the lens of intercellular communication. Most mechanistic studies in this field still rely on two-dimensional OS cell lines, simplified co-culture systems, or immunodeficient xenograft models. Although these approaches have been valuable for dissecting individual pathways, they only partially reproduce the mineralized extracellular matrix, spatial immune organization, vascular heterogeneity, organ-specific metastatic niches, and biomechanical constraints of human OS.

Evidence on EVs is also difficult to compare across studies because vesicle populations are heterogeneous and isolation, quantification, and characterization methods vary substantially. In several experimental settings, vesicle doses may not reflect physiological exposure, making it difficult to determine which cargo species are truly dominant *in vivo*. Similarly, many immune studies are based on bulk transcriptomic or immunohistochemical analyses, which can mask spatially restricted macrophage, dendritic-cell, T-cell, and myeloid-suppressor states. More representative systems, including patient-derived organoids, orthotopic immunocompetent models, spatially resolved functional assays, and longitudinal paired primary and metastatic samples, remain insufficiently used.

Clinical translation is further limited by small patient cohorts, histologically mixed sarcoma trials, inconsistent biomarker selection, heterogeneous endpoints, and limited serial sampling before and after treatment. At present, longitudinal patient-level evidence directly linking adaptive TME communication to treatment failure remains limited. Likewise, direct comparative evidence demonstrating that disruption of communication networks is more effective than targeting tumor-intrinsic mechanisms is still lacking.

## 9 Discussion

This review frames OS as a communication-driven malignancy in which tumor progression emerges from complex and multilayered signaling exchanges between malignant, stromal, immune, and bone-resident cells. Rather than functioning as autonomous units, OS cells continuously engage in bidirectional interactions mediated by soluble factors, EVs, direct cell-cell contacts, and mechanical cues. These networks coordinate proliferation, immune evasion, metastatic dissemination, and therapeutic resistance, supporting a shift from a tumor-centric model toward a systems-oriented understanding of OS biology.

Immune cell–mediated communication represents a central regulatory axis. TAMs, MDSCs, dysfunctional DC subsets, and exhausted T-cell populations operate within interconnected feedback loops that reinforce immune suppression. The plasticity and spatial heterogeneity of these populations likely explain the limited efficacy of immune checkpoint blockade observed in clinical trials. These findings suggest that effective immunotherapy in OS will require coordinated reprogramming of multiple immune compartments rather than isolated checkpoint inhibition.

Beyond immune regulation, EVs and soluble mediators extend the tumor’s influence locally and systemically, shaping angiogenesis, metabolic adaptation, and pulmonary pre-metastatic niche formation. In parallel, direct physical communication through gap junctions and tunneling nanotubes enhances intratumoral coordination and stress adaptation, reinforcing heterogeneity and survival under adverse conditions. Together, these mechanisms form an integrated communication architecture that enables collective malignant behavior. More broadly, stress-adaptation readouts are increasingly interpreted as context-dependent translational markers rather than isolated mechanistic surrogates (245, 246). Similar caution applies to angiogenic and microenvironmental indicators, which require disease-specific interpretation before clinical translation (247, 248).

Importantly, the profound genomic instability characteristic of OS may serve as both a driver and a consequence of this communicative plasticity. Structural rearrangements and chromosomal instability not only generate cellular heterogeneity but also influence inflammatory signaling, EV production, and immune modulation, linking intrinsic genomic chaos to extrinsic microenvironmental remodeling.

Despite substantial mechanistic advances, translation into durable clinical benefit remains limited. Many proposed targets derive from preclinical systems that incompletely recapitulate the spatial, biomechanical, and immune complexity of human bone tumors. Furthermore, signaling redundancy and compensatory pathway activation present major obstacles to single-agent interventions.

From a therapeutic standpoint, targeting intercellular communication networks offers a rational strategy to overcome these limitations. However, given the adaptive and interconnected nature of OS signaling circuits, successful interventions will likely require biomarker-guided, combinatorial approaches capable of simultaneously modulating immune polarization, angiogenic signaling, stromal interactions, and EV-mediated communication. Comparable caution is also warranted for immune-checkpoint markers (249, 250). Molecular surveillance workflows likewise require integration with histopathologic and clinical context rather than assay positivity alone (251–253).

In summary, conceptualizing OS as a network-based disease provides a unifying framework that integrates genomic instability, immune dysregulation, and microenvironmental remodeling into a coherent biological model. Future progress will depend on translating high-resolution molecular insights into clinically actionable strategies aimed not merely at inhibiting tumor cells, but at dismantling the communication circuits that sustain malignant adaptation.
